# A monoallelic variant in *CCN2* causes an autosomal dominant spondyloepimetaphyseal dysplasia with low bone mass

**DOI:** 10.1038/s41413-024-00364-2

**Published:** 2024-10-16

**Authors:** Shanshan Li, Rui Shao, Shufa Li, Jiao Zhao, Qi Deng, Ping Li, Zhanying Wei, Shuqin Xu, Lin Chen, Baojie Li, Weiguo Zou, Zhenlin Zhang

**Affiliations:** 1 Department of Osteoporosis and Bone Diseases, Shanghai Clinical Research Center of Bone Diseases, Shanghai Jiao Tong University of Medicine Affiliated Sixth People’s Hospital, Shanghai, China; 2 Department of Orthopedic Surgery and Shanghai Institute of Microsurgery on Extremities, Shanghai Jiao Tong University of Medicine Affiliated Sixth People’s Hospital, Shanghai, China; 3 Department of Endocrinology and Metabolism, The Affiliated Hospital of Qingdao University, Qingdao, Shandong China; 4 Key Laboratory for the Genetics of Developmental and Neuropsychiatric Disorders, Bio-X Institutes, Ministry of Education, Shanghai Jiao Tong University, Shanghai, China; 5 Department of Wound Repair and Rehabilitation, State Key Laboratory of Trauma, Burns and Combined Injury, Trauma Center, Research Institute of Surgery, Daping Hospital, Army Medical University, Chongqing, China; 6 Key Laboratory of RNA Innovation, Science and Engineering, CAS Center for Excellence in Molecular Cell Science, Shanghai Institute of Biochemistry and Cell Biology, University of Chinese Academy of Sciences, Chinese Academy of Sciences, Shanghai, China; 7 Hainan Academy of Medical Sciences, Hainan Medical University, Hainan, China

**Keywords:** Bone, Metabolic bone disease

## Abstract

Cellular communication network factor 2 (CCN2) is a secreted extracellular matrix-associated protein, and its aberrantly increased expression has been implicated in a diversity of diseases involving pathological processes of fibrosis, chronic inflammation, or tissue injury, which has promoted the evaluation of CCN2 as therapeutic targets for multiple disorders. However, human phenotypes associated with CCN2 deficiency have remained enigmatic; variants in *CCN2* have not yet been associated with a human phenotype. Here, we collected families diagnosed with spondyloepimetaphyseal dysplasia (SEMD), and screened candidate pathogenic genes for families without known genetic causes using next-generation sequencing. We identified a monoallelic variant in signal peptide of *CCN2* (NM_001901.2: c.65 G > C [p.Arg22Pro]) as the cause of SEMD in 14 subjects presenting with different degree of short stature, premature osteoarthritis, and osteoporosis. Affected subjects showed decreased serum CCN2 levels. Cell lines harboring the variant displayed decreased amount of CCN2 proteins in culture medium and an increased intracellular retention, indicating impaired protein secretion. And the variant weakened the stimulation effect of CCN2 on osteogenesis of bone marrow mesenchymal stem cells. Zebrafish *ccn2a* knockout model and osteoblast lineage-specific *Ccn2*-deficient mice (*Ccn2*^*fl/fl*^*;Prx1*^*Cre*^) partially recapitulated the phenotypes including low bone mass observed in affected subjects. Pathological mechanism implicated in the skeletal abnormality in *Ccn2*^*fl/fl*^*;Prx1*^*Cre*^ mice involved decreased bone formation, increased bone resorption, and abnormal growth plate formation. Collectively, our study indicate that monoallelic variants in *CCN2* lead to a human inherited skeletal dysplasia, and highlight the critical role of CCN2 in osteogenesis in human.

## Introduction

Skeletal dysplasia is a group of human genetic disorders primarily involved musculoskeletal system.^1^ Spondyloepimetaphyseal dysplasia (SEMD) is among the most complicated subgroups of skeletal dysplasia, and is a collective term for clinically and genetically heterogeneous disorders marked by vertebral, epiphyseal, and metaphyseal abnormality.^1,2^ Bone deformity and premature osteoarthritis are usually the prominent clinical features of SEMD. In cases that SEMD is observed as a part of a syndrome, extraskeletal symptoms such as mental retardation, hypotonia, hypotrichosis, myopia, and atrophic scarring can also be predominant features. More than 20 subgroups of SEMD including Strudwick type, Aggrecan type, Matrilin type, Missouri type, and short limb-abnormal calcification type with different modes of inheritance have been described so far.^1^ No common molecular pathway has been identified yet in SEMD. Nevertheless, encoding proteins of a considerable part of established culprit genes are found to be components of extracellular matrix (ECM) or involved in processes of ECM metabolism.^3–9^ In these cases, variants in the pathogenic genes result in, for example, abnormal effects of protein on ECM, reduced protein secretion, or endoplasmic reticulum stress due to misfolded protein, leading to defects in the ECM structural framework or mediated biological signals necessary for skeletal growth and homeostasis.^10,11^ Substantial progresses have been made with respect to the genetic research in SEMD, however, the genetic heterogeneity as well as the paucity of families with multiple affected subjects have hindered the identification of new genetic causes for SEMD, hampering the molecular diagnosis and genetic counseling for a considerable number of affected families in clinical practice.

Cellular communication network factor 2 (CCN2), also known as connective tissue growth factor, is among established matricellular proteins, a group of non-structural proteins presented in ECM, and serves primarily regulatory roles.^12–14^ CCN2 was initially found to be mitogenic and chemotactic for fibroblast-like cells, and early studies were largely focused on its pathological significance in fibrotic disorders.^15–17^ In recent years, mounting evidence drawn from animal models and human patients have suggested the implication of aberrant CCN2 in a remarkable diversity of diseases besides organ fibrosis, such as diabetic nephropathy and retinopathy, carcinogenesis and tumor development, atherosclerosis, and arthritis, most of which are verified related to pathological processes of chronic inflammation or tissue injury.^18,19^ Emerging studies describe CCN2 as promising diagnostic or prognostic markers for these diseases.^18^ Moreover, potential therapeutic effect and safety of several drugs targeting CCN2 (i.e., FG-3019, OLX-10010, EXC-001) have been evaluated in a series of disorders, such as diabetic nephropathy, idiopathic pulmonary fibrosis, pancreatic cancer, and results drawn from these clinical trials are highly encouraging.^20–23^ In addition to the involvement of CCN2 in pathological conditions, it displays multiple physiological functions, and is involved in such as central nervous system development, spinal cord regeneration, and wound healing.^17,19,24^ Despite the many pivotal functions of CCN2 in both pathological and physiological situations, human phenotypes associated with CCN2 deficiency remain to be elucidated, and no human monogenic disorder has been associated with variants in *CCN2* gene to date.

In present study, we collected families diagnosed with SEMD, and screened candidate pathogenic genes for families without known genetic causes using next-generation sequencing. Mechanism studies were further performed to explore underlying pathogenesis of the candidate genes.

## Results

### *CCN2* variant shows complete cosegregation with clinical traits in a large family with SEMD

**Fig. 1:**
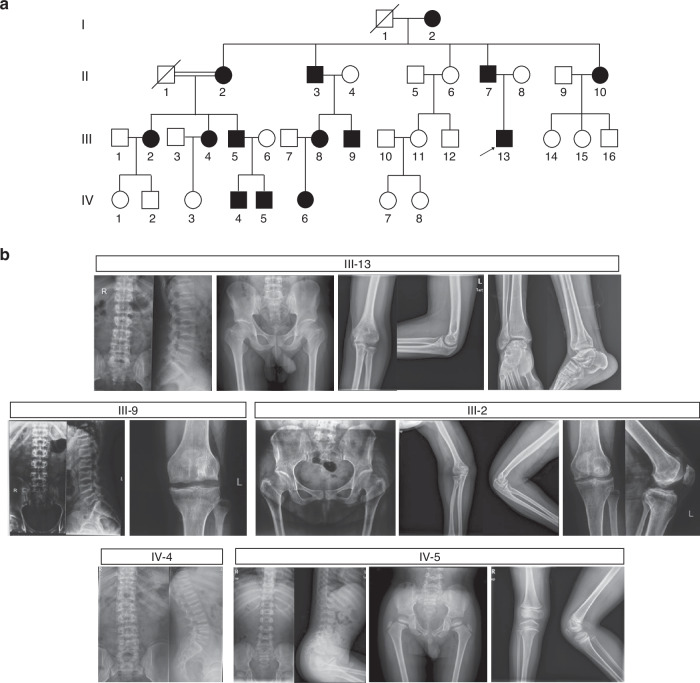
Pedigree and radiographical features of a large Chinese family with spondyloepimetaphyseal dysplasia (SEMD). **a** Pedigree of the family with SEMD. Squares and circles indicate males and females, respectively. Black symbols present affected individuals, and slashes deceased individuals. Arrow indicates the proband. **b** Radiographic abnormalities of subjects with SEMD. III-13: individual at age 25.7 years. Spine lateral shows platyspondyly and biconcave deformities of the central-posterior portion of vertebral bodies, with irregular sclerotic end plates. Pelvis shows mildly narrow iliac wings with irregular acetabulum and femoral head. Short and broad femoral necks are noted. Elbow and ankle joint show irregular proximal humerus and distal tibiae and fibulae, as well as hyperostosis. The joint spaces are preserved. III-9: individual at age 28.9 years. Platyspondyly with irregular sclerotic end plates, and biconcave vertebral bodies are noted. Knee joint shows mildly decreased joint spaces, irregular articular surface, osteophyte formation, together with sclerotic and cystic changes. III-2: individual at age 44.6 years. Femoral necks are short and broad, with irregular acetabuli and femoral head. Degenerative changes, including narrow joint cavity, hyperostosis, subchondral cysts and bone sclerosis are noted in hip, elbow and knee joints. IV-4: individual at age 15.1 years. Spine lateral shows flat vertebral bodies, with irregular epiphyses and mild scoliosis. IV-5: individual at age 4.6 years. Distinctive bullet-shaped appearance vertebral bodies and irregular epiphyses are noted. Pelvis and knee joint show irregular epiphyses and metaphyses of the long bones, together with acetabular abnormalities

We detected a monoallelic variant (c.65 G > C, p.Arg22Pro) in *CCN2* (NM_001901.2) in a four-generation Han Chinese family with an autosomal dominant form of SEMD, and a total of 14 subjects were affected (Fig. 1a). Affected individuals appeared asymptomatic at birth. The earliest changes were noticed at early childhood, and manifested as an abnormal walking or running gait. The majority of affected subjects suffered from limited flexion of knee, ankle, and elbow joint since their childhood. Joint pain and swelling in lower limbs developed around adolescence, gradually getting worse as they aged. The affected subjects also showed mild-to-severe disproportionate short stature with relative short lower limbs.^25^^,^^26^ Overt extraskeletal abnormalities were not observed. Radiographs prior to epiphyseal fusion displayed dysplastic epiphyses and irregular metaphyses of long bones, as well as bullet-shaped appearance of vertebral bodies with irregular epiphyses; while after epiphyseal fusion, exhibited platyspondyly with biconcave deformities of the central-posterior portion of vertebral bodies, irregular sclerosis of endplates, dysplasia acetabulum and proximal femur, and dysplasia of elbow and ankle joint with osteophyte formation, and in some subjects, showed obvious premature degenerative changes in weight-bearing joints that resembled radiographic features of osteoarthritis at elder ages, including decreased joint spaces, irregular articular surface, hyperostosis, subchondral cysts and bone sclerosis (Fig. 1b). Bone mineral density (BMD) measurement, which was qualified using dual-energy X-ray absorptiometry, showed osteoporosis or osteopenia in 6 of 8 adults.^27^ Detailed clinical features and radiographic findings were shown in Table 1, Table 2, and Supplementary Note. No significant abnormalities were detected in routine biochemical tests.

**Table 1:** Clinical features of subjects with the *CCN2* variant

Subject, Age (year)/Sex^a^	Waddling gait	Limited joint flexion	Limited joint extension	Joint swelling and pain	Height (SD)^b^/cm	Arm span/cm	U/L ratio	Head circumference/cm	Other
I-2, 80.2/F	+	N/A	N/A	+	127.0 (−4.16)	N/A	N/A	N/A	kyphosis
II-2, 70.0/F	+	+	+	+	144.0 (−1.38)	152.0^c^	1.0	55.0	-
II-3, 67.2/M	+	N/A	N/A	+	148.0 (−2.74)	N/A	N/A	N/A	N/A
II-7, 56.9/M	+	+	+	+	145.0 (−3.43)	158.5^c^	1.1	56.0	kyphosis, IJE, genu varum
II-10, 53.7/F	+	+	+	+	140.1 (−2.71)	148.0^c^	1.1	55.5	-
III-2, 44.6/F	+	+	-	+	138.2 (−3.46)	152.0	1.1	58.2	genu valgum
III-4, 41.1/F	+	+	-	+	140.2 (−3.11)	149.0	1.1	56.5	genu varum
III-5, 39.5/M	+	+	-	+	154.7 (−2.26)	165.5	1.1	59.0	IJE
III-8, 33.3/F	+	+	-	+	153.4 (−0.75)	161.5	1.0	54.0	joint stiffness, genu varum
III-9, 28.9/M	+	+	+	+	161.6 (−1.15)	177.0^c^	1.0	58.0	twice KAS
III-13, 25.7/M	+	+	-	+	154.9 (−2.23)	173.0	1.1	55.0	-
IV-4, 15.1/M	+	+	-	+	162.5 (−1.12)	175.0	1.2	61.0	DCF
IV-5, 4.6/M	+	-	-	-	107.8 (0.02)	106.5	1.4	53.0	DCF
IV-6, 6.3/F	+	-	-	-	118.6 (−0.17)	120.5	1.1	49.0	-

*F* female, *M* male, *SD* standard deviation, *U/L ratio* upper segment/lower segment, *IJE* interphalangeal joint enlargement, *KAS* knee arthroscopic surgeries, *DCF* delayded closure of the fontanel, *N/A* not available, + denotes yes, – denotes unobserved

^a^Roman numerals indicate generation, and arabic numbers indicate the order of offspring within a generation

^b^SD, deviation from the mean height of the Chinese population^25^^,^^26^

^c^II-2, II-7, II-10, and III-9 have limited extension of elbow joints

**Table 2:** Radiographic features and bone mineral density of subjects with the *CCN2* variant

Subject, Age (year)/ Sex^a^	Radiographic features					Bone mineral density^b^					
	Spine deformity	Abnormal metaphyses	Irregular epiphyses	OA like changes	Other	L1-4/ (g/cm^2^)	Z-scores/ T-scores	Neck/ (g/cm^2^)	Z-scores/ T-scores	Total/ (g/cm^2^)	Z-scores/ T-scores
II-2, 70.0/F	platyspondyly	+	N/A	+	SFN	N/A	N/A	N/A	N/A	N/A	N/A
II-7, 56.9/M	platyspondyly	+	N/A	+	SFN	0.734	−2.4/−2.9	0.585	−2.2/−3.0	0.615	−2.6/−2.9
II-10, 53.7/F	platyspondyly	+	N/A	+	SFN	0.767	−1.2/−2.9	0.689	−0.6/−2.0	0.691	−1.1/−2.2
III-2, 44.6/F	platyspondyly, mild scoliosis	+	N/A	+	SFN	0.875	−2.4/−2.0	0.638	−2.4/−2.4	0.756	−1.8/−1.7
III-4, 41.4/F	N/A	N/A	N/A	N/A	N/A	0.842	−1.7/−2.3	0.679	−1.6/−2.1	0.800	−0.9/−1.3
III-5, 39.5/M	N/A	+	N/A	+	N/A	0.701	−2.7/−3.2	0.633	−2.2/−2.7	0.678	−2.3/−2.4
III-8, 33.3/F	platyspondyly	+	N/A	-	-	0.945	−0.7/−1.4	0.640	−1.9/−2.4	0.632	−2.2/−2.6
III-9, 28.9/M	platyspondyly, ISEP	+	N/A	+	SFN	1.094	0.1/0.1	0.849	−1.0/−1.0	0.893	−0.8/−0.8
III-13, 25.7/M	platyspondyly, ISEP	+	N/A	+	SFN	1.053	−0.3/−0.2	0.803	−1.5/−1.3	0.829	−1.3/−1.2
IV-4, 15.1/M	platyspondyly, mild scoliosis	+	+	-	SFN	0.861	N/A	0.797	N/A	0.798	N/A
IV-5, 4.6/M	BSVB	+	+	-	IAPF	0.416	N/A	0.576	N/A	0.553	N/A
IV-6, 6.3/F	BSVB	+	+	-	IAPF	0.574	N/A	0.537	N/A	0.467	N/A

*F* female, *M* male, *OA* osteoarthritis, *L1-4* lumber spine 1–4, *Neck* femoral neck, *Total* total hip, *ISEP* irregular sclerotic end plates, *BSVB* bullet-shaped vertebral bodies, *SFN* short femoral necks, *IAPF* irregular acetabulum and proximal femur, *N/A* not available, + denotes yes, – denotes unobserved

^a^Roman numerals indicate generation, and arabic numbers indicate the order of offspring within a generation

^b^Bone mineral density was qualified using dual-energy X-ray absorptiometry, and Z-scores/T-scores were calculated based on equipment-specific references^27^

**Fig. 2:**
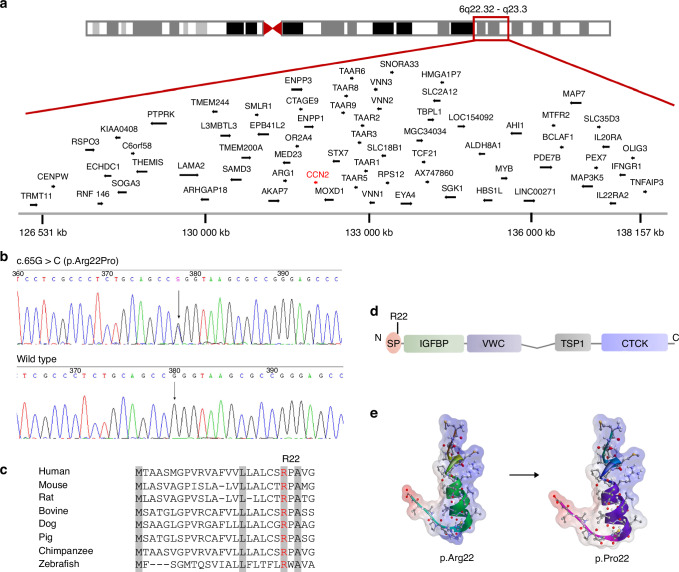
Identification of a pathogenic variant in *CCN2* in the family with spondyloepimetaphyseal dysplasia (SEMD). **a** Schematic representation of chromosome 6 and a single overlapping region on chromosome 6q22.32–q23.3 identified by linkage analysis. **b** Sequencing chromatogram of a missense variant (c.65 G > C, arrow) in exon 1 in *CCN2*. The variant shows segregation with SEMD in all available family members. The base change predicts the replacement of the amino acid arginine in codon 22 with proline (p.Arg22Pro). **c** Alignments of the region of CCN2 from multiple species, showing the altered residue in red. Highly conserved amino acids are indicated in gray. **d** Schematic diagrams of protein domain organization of CCN2, along with the position of the identified variant. The molecular structure of CCN2 consists of a signal peptide (SP) and four conserved domains with sequence homologies to Insulin-like growth factor binding protein (IGFBP), von Willebrand factor type C domain (VWC), Thrombospondin type 1 repeat (TSP-1), and a carboxy-terminal cystine knot domain (CTCK). The amino acid residue Arg22 is located in the SP. **e** Structural model of SP of CCN2. Positive and negative charged regions are indicated in blue and red, respectively, and uncharged area is shown in white. The mutant residue Arg22 results in a decrease in positive charge region

To identify genetic cause of the large Chinese family with SEMD, we used a combination of genome-wide linkage scan, whole-exome sequencing (WES), and Sanger sequencing. Genome-wide linkage analysis was first applied in 18 family members, including I-2, II-2, II-6, II-7, II-9, II-10, III-1, III-2, III-3, III-4, III-5, III-6, III-13, III-14, III-15, IV-2, IV-3, and IV-4. Linkage data was analyzed with a dominant model of inheritance, and revealed a single significant candidate region on chromosome 6q22.32-q23.3, flanked by markers kgp22768966 and rs9389536, with a maximum LOD score of 3.5 (Fig. 2a, Fig. S1). And we did not identify any genes known to cause SEMD in this region. WES was subsequently conducted in four affected (II-2, III-9, III-13, and IV-4) and two unaffected individuals (II-6 and III-15), and revealed 33 variants in 27 genes that were co-segregating with the clinical trait among these six subjects (Table S1). Meanwhile, eight variants were located in the linked region, and two variants of them, respective in *CCN2* and *FBXO30* gene, would potentially lead to coding-protein change. Sanger sequencing confirmed the existence of both variants, and primers used for PCR and direct sequencing are 5’-AGGTGGGGAGGAATGCGAGGA-3’ (forward), 5’-GCCGTCCAGCACGAGGCTCAC-3’ (reverse) for the variant in *CCN2*, and 5’-ATGGCTCTGAATTTGTGCAGT-3’ (forward), 5’-ACTGAGCGTAGTGAACGTCCT-3’ (reverse) for the variant in *FBXO30*. However, only the variant (c.65 G > C, p.Arg22Pro) in *CCN2* (NM_001901.2) showed complete cosegregation with clinical traits in all available family members (*n* = 28), and was considered to be the excellent candidate causative variant for the disorder (Fig. 2b). To our knowledge, the variant in *CCN2* is absent in current public databases of human sequence variants, including the 1000 Genomes Project, the Single Nucleotide Polymorphism database, the ESP, and the ExAC database, and it is also absent in the 750 control samples (1 500 alleles) enrolled in our study. The amino acid Arg22 residue is located in the signal peptide of CCN2, and is highly conserved across various species (Fig. 2c, d). Structural analysis showed elevated folding free energy leading to conformational changes in the mutant CCN2. Meanwhile, decreased isoelectric point of the signal peptide due to the mutant residue was indicated, which would probably impair the process of protein secretion (Fig. 2e).

### *CCN2* variant impairs CCN2 protein secretion and serum CCN2 levels in affected subjects are decreased

**Fig. 3:**
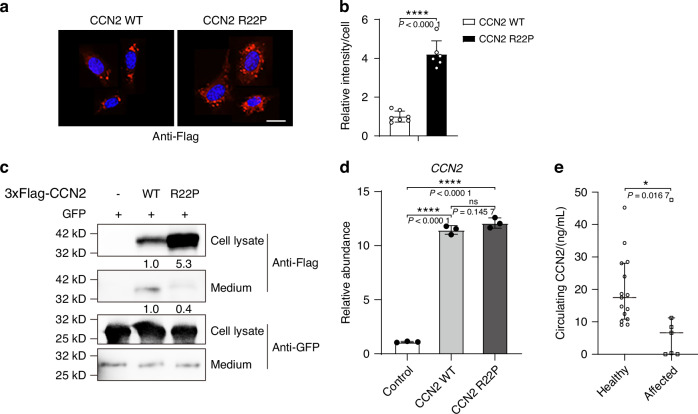
*CCN2* R22P variant impairs CCN2 protein secretion. **a**, **b** Immunofluorescence analyses of intracellular distribution of Flag-tagged hCCN2 protein in C3H10T1/2 cells infected with plasmids encoding Flag-tagged wild-type or mutant hCCN2 (p.Arg22Pro) after 48 h. Scale bar = 10 μm. Data are represented as mean ± SD (*n* = 7 for wild-type, *n* = 6 for mutant). Student’s *t* test was used. ****P < 0.000 1*.* **c** Western blot analysis of Flag-tagged hCCN2 protein abundance in the cell lysates and culture medium of HEK293T cells transfected with indicated plasmids after 48 h. The relative expression of hCCN2 was monitored with the use of monoclonal antibody to Flag, showing bands of the expected size (~40 kD). **d** RT-PCR of total mRNA of CCN2 from HEK293T cells transfected with indicated plasmids after 48 h. Data are represented as mean ± SD, normalized by the housekeeping Gapdh. Student’s *t* test was used. ****P < 0.000 1; ns, not significant. **e** Serum CCN2 levels in affected subjects with SEMD (*n* = 7) and healthy controls (*n* = 15), data are represented as median with interquartile range. Mann–Whitney test was used. *P < 0.05

To assess whether the variant affect CCN2 secretion, we overexpressed Flag-tagged human wild-type or mutant (p.Arg22Pro) CCN2 in cell lines using plasmid vector, and respectively collected the culture medium and cells after transfection of 48 h. We detected a lower amount of CCN2 protein in the culture medium while an increased intracellular retention of the protein in cells transfected with the mutant CCN2, using immunofluorescence and western blot analysis, compared with cells transfected with the wild-type (Fig. 3a–c). We then performed RT-PCR to detect the levels of CCN2 mRNA, and found that the mutant transfected cells had similar increased levels of CCN2 mRNA with those of wild-type transfected cells (Fig. 3d). These findings indicated that the reduced secretion of CCN2 protein was most likely attributed to the detrimental effect of the variant on protein secretion. Consistent with the discoveries in the cell lines, serum CCN2 levels of affected subjects were significantly lower than those of healthy controls (Fig. 3e).

### *CCN2* variant weakens its stimulation effect on osteogenesis of bone marrow mesenchymal stem cells

**Fig. 4:**
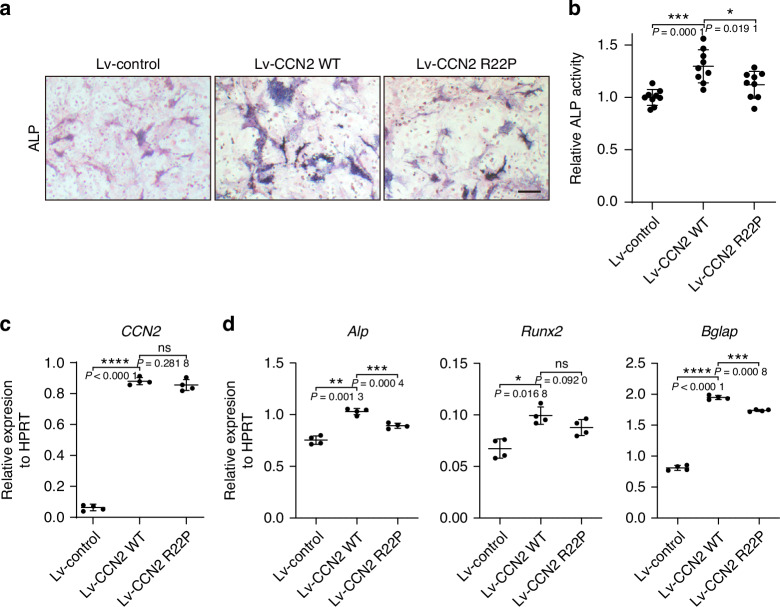
*CCN2* variant weakens its stimulation effect on osteogenesis of bone marrow mesenchymal stromal cells (BMSCs). **a** ALP staining results of osteoblast differentiation of BMSCs infected with indicated lentivirus overexpressing wild-type or mutant hCCN2 (p.Arg22Pro), and ZsGreen lentivirus was used as a control (*n* = 9). Scale bar = 10 μm. **b** ALP quantification results of BMSCs infected with indicated lentivirus at day 7. Data are represented as mean ± SD. Student’s *t* test was used (*n* = 9). *P < 0.05; ***P < 0.001. RT-PCR analysis of *CCN2* (**c**) and *Alp*, *Runx2*, *Bglap* (**d**) expression at day 7 during BMSCs differentiation. Data are represented as mean ± SD. Student’s *t* test was used (*n* = 4). *P < 0.05; **P < 0.01; ***P < 0.001; ****P < 0.000 1; ns, not significant

To further clarify the functional consequence of *CCN2* variant, we overexpressed human wild-type or mutant CCN2 in mice bone marrow mesenchymal stem cells (BMSCs) using lentivirus. As shown in Fig. 4a, b, the osteoblast differentiation of the mutant CCN2 infected BMSCs were lower than those of wild-type infected cells, as determined by the ALP staining and activity quantification. Although the expression level of CCN2 of mutant infected cells increased to a similar extent to those of wild-type infected cells (Fig. 4c), the osteoblast differentiation marker genes *Alp*, *Runx2*, and *Bglap* increased to a less extent (Fig. 4d). These results indicated that CCN2 played a role in promoting osteoblast differentiation, and the identified variant in CCN2 reduce its effectiveness in this process.

### *Ccn2a* knockdown zebrafish show severe craniofacial skeleton dysplasia and decreased bone mass, and are rescued by *ccn2a* mRNA overexpression

**Fig. 5:**
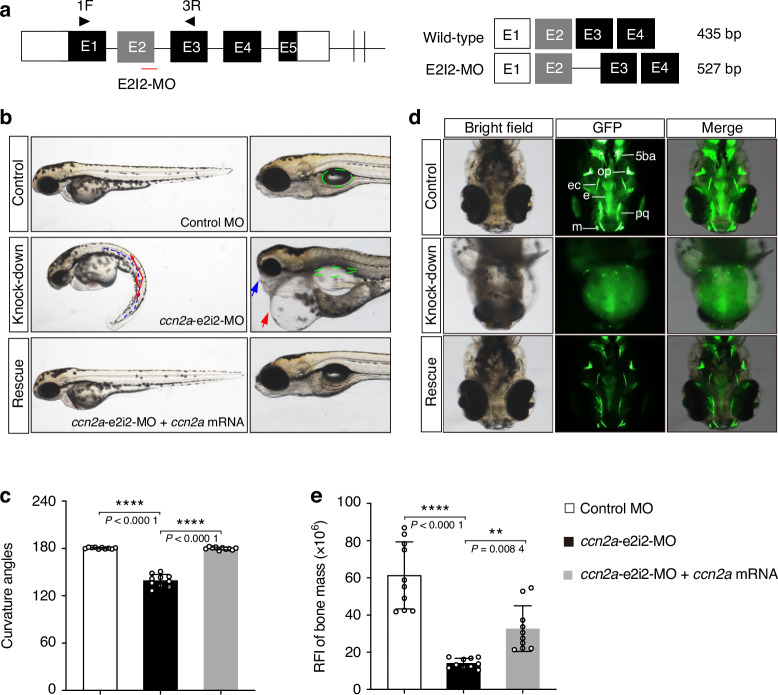
Overexpression of *ccn2a* mRNA rescues morphological abnormalities and craniofacial phenotypes in *ccn2a* knockdown zebrafish. **a** Zebrafish *ccn2a* gene is targeted by specific morpholino antisense to prevent proper splicing of exon 2 (E2I2-MO). Primers 1 F and 3 R interrogate the presence of wild type transcripts or those in which intron 2 has been inserted. The diagram is a schematic depiction of the intron 2 inserted transcript in the E2I2-MO injected embryos (527 bp) as compared with control MO injected embryos (435 bp). **b** Representative lateral views of zebrafish at 2 days post-fertilization (dpf) injected with either control MO or *ccn2a* MO with or without *ccn2a* mRNA. The *ccn2a* morphants display curved body axis (blue dotted line), small eyes, pericardial oedema (red arrow), jaw defects (blue arrow), and non-inflated swim bladder (green circled area). Co-injection of nonmutant zebrafish *ccn2a* mRNA rescue the gross morphology in *ccn2a* morphants. **c** Quantification of the average curvature angle of zebrafish in each group at 2-dpf. Data are represented as mean ± SD (*n* = 10). One way ANOVA with Bonferroni’s post hoc multiple comparisons was used. ****P < 0.000 1. **d** Representative ventral view of the craniofacial skeleton of zebrafish in each group at 5-dpf labeled with calcein. 5ba, fifth branchial arch; op, opercular bone; ec, ectopterygoid; e, ethmoid plate; pq, palatoquadrate; m, Meckel’s cartilage. Co-injection of nonmutant zebrafish *ccn2a* mRNA significantly rescued head skeletal malformation in *ccn2a* morphants. **e** Quantification of the relative fluorescence intensity (RFI) of craniofacial skeleton bone mass of zebrafish in each group at 5-dpf (*n* = 10). Data are represented as mean ± SD. One way ANOVA with Bonferroni’s post hoc multiple comparisons was used. **P < 0.01; ****P < 0.000 1

Zebrafish exhibit a simple cartilage and bone development pattern which is most highly conserved among all vertebrates during evolution. Genetic studies have revealed similarities in developmental mechanisms underlying skeletal morphogenesis between zebrafish and human. These findings make zebrafish an excellent model to study molecular basis of cartilage and bone development.^28,29^ The protein sequence of zebrafish homolog, *ccn2a*, is 78.7% identical to human CCN2 protein. Therefore, we designed and validated specific morpholino oligonucleotides (MO) to knockdown the *ccn2a* in zebrafish to characterize the functional consequences of CCN2 deficiency in skeletal development (Fig. 5a, Fig. S2). The exon 2-intron 2 splice *ccn2a* MO (*ccn2a*-e2i2-MO) or the standard control MO was microinjected into fertilized one-cell stage embryos. And the zebrafish injected with *ccn2a*-e2i2-MO showed overt gross morphologic abnormalities at 2 days post-fertilization (dpf), including remarkable curved body axis, jaw defects, small eyes, pericardial oedema, and non-inflated swim bladder (Fig. 5b, c, Fig. S3). To further visualize the skeletal structures in zebrafish embryos, we performed calcein staining.^30^ At 5-dpf, fluorescent signals of well-developed craniofacial cartilage structures became apparent, and the *ccn2a*-e2i2-MO injected larvae showed severe dysplasia of craniofacial skeleton (Fig. 5d, Fig. S3). The craniofacial defects included the absence of Meckel’s cartilage, palatoquadrate, and fifth branchial arch, and the dysmorphic opercular bone, ectopterygoid, and ethmoid plate. The amount of stained mineralized tissue in mutant morphants was markedly reduced comparing with that of control, and quantification of the relative fluorescence intensity (RFI) of head skeleton bone mass showed significant decrease in *ccn2a* morphants (Fig. 5e). To confirm the skeleton dysplasia in *ccn2a*-e2i2-MO injected zebrafish were specially caused by *ccn2a* knockdown, we performed rescue experiments by co-injecting *ccn2a*-e2i2-MO with *ccn2a* mRNA, and observed that overexpression of *ccn2a* mRNA significantly rescued the skeleton malformation in *ccn2a* morphants (Fig. 5d, e). These findings suggest that *ccn2a* is essential for skeletal development in zebrafish.

### Osteoblast lineage-specific *Ccn2*-deficient mice present with decreased bone mass

**Fig. 6:**
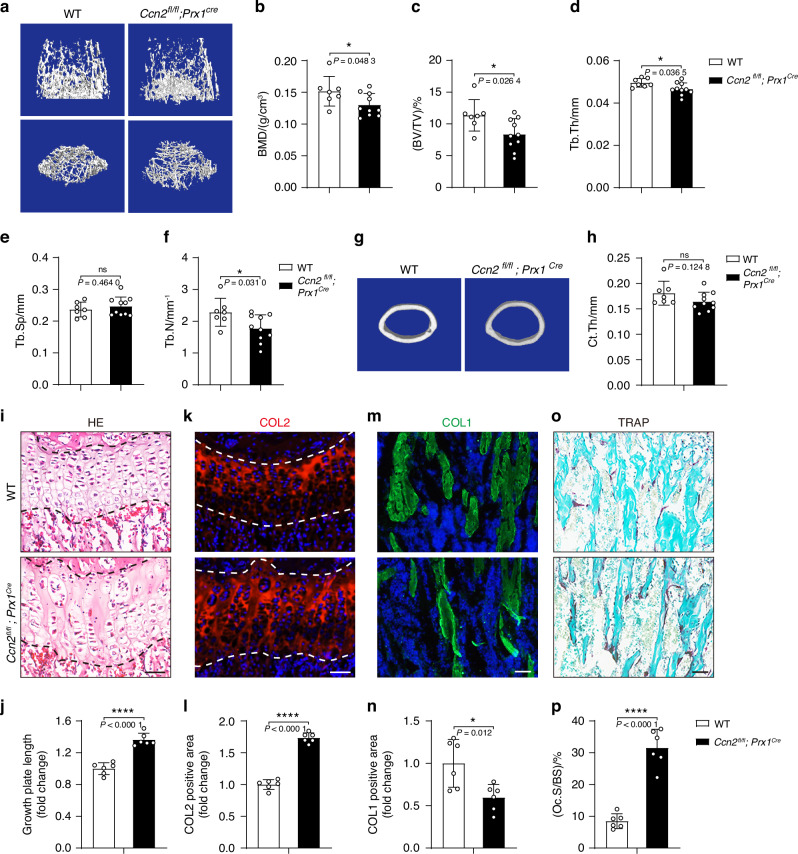
Deletion of *CCN2* in mesenchymal progenitor cells leads to decreased bone mass. **a**–**f** Micro-CT image of trabecular bones of distal femurs isolated from 6-week-old wild-type and *Ccn2*^*fl/fl*^*;Prx1*^*Cre*^ mice, and analysis for bone mineral density (BMD), bone volume per tissue volume (BV/TV), trabecular thickness (Tb.Th), trabecular spacing (Tb.Sp), and trabecular number (Tb.N). Data are represented as mean ± SD (*n* = 7 for wild-type, *n* = 10 for *Ccn2*^*fl/fl*^*;Prx1*^*Cre*^)*.* Student’s *t* test was used. *P < 0.05; ns, not significant*.* **g**, **h** Micro-CT image of cortical bones of mid diaphysis of femurs isolated from 6-week-old mice, and analysis for cortical thickness (Ct.Th). Data are represented as mean ± SD (*n* = 7 for wild-type, *n* = 10 for *Ccn2*^*fl/fl*^*;Prx1*^*Cre*^). Student’s *t* test was used. ns, not significant. **i**, **j** Histology of growth plate in femurs of 6-week-old mice. The sections are stained with H/E. Scale bar = 50 μm. Data are represented as mean ± SD (*n* = 6). Student’s *t* test was used. **** *P <* 0.000 1. **k**, **l** Immunofluorescence analysis of Col2 of growth plate in femurs of 6-week-old mice. Scale bar = 50 μm. Data are represented as mean ± SD (*n* = 6). Student’s *t* test was used. ****P < 0.000 1*.* **m**, **n** Immunofluorescence analysis of Col1 in femurs of 6-week-old mice. Scale bar = 50 μm. Data are represented as mean ± SD (*n* = 6). Student’s *t* test was used.*P < 0.05*.* **o**, **p** TRAP staining in femurs of 6-week-old mice. Scale bar = 50 μm. Data are represented as mean ± SD (*n* = 6). Student’s *t* test was used. ****P < 0.000 1

To understand the underlying molecular mechanism for the skeletal phenotypes of affected subjects with CCN2 deficiency, we determined whether the defects in skeletal development were caused by requirement of CCN2 in early mesenchymal progenitor cells. We knockout *CCN2* in mesenchymal progenitors in mice (*Ccn2*^*fl/fl*^*;Prx1*^*Cre*^) by crossing *Ccn2*^*fl/fl*^ mice with *Prx1*^*Cre*^ mice, and the latter is a transgenic line that expressed Cre recombinase in a subset of early limb bud mesenchyme.^31^ To clarify the effect of CCN2 in skeletal system, we performed micro-CT to analyze the limbs isolated from the 6-week-old *Ccn2*^*fl/fl*^*;Prx1*^*Cre*^ mice and corresponding control littermates. The *Ccn2*^*fl/fl*^*;Prx1*^*Cre*^ mice displayed decreased femoral trabecular bone mass relative to their control littermates (Fig. 6a–h), with a reduction in BMD, bone volume fraction (BV/TV), thickness (Tb.Th) and number (Tb.N). And the measurements including trabecular separation (Tb.Sp) and cortical thickness (Ct.Th) of *Ccn2*^*fl/fl*^*;Prx1*^*Cre*^ mice were similar to age-matched control littermates. To further understand how *CCN2* deletion in mesenchymal progenitors caused the bone abnormality, we performed morphological analysis of femurs from 6-week-old mice. Hematoxylin and eosin (H/E) staining displayed distorted and enlarged of growth plate as shown in Fig.  6i, j. And immunofluorescence analysis for cartilage anabolism markers showed an expansion of Col2-positve area (Fig. 6k, l), and the number of the Col1α1-positive area were reduced in the *Ccn2*^*fl/fl*^*;Prx1*^*Cre*^ mice (Fig. 6m, n). And the bone resorption as detected through TRAP staining showed an increased TRAP-positive area (Fig. 6o, p). These results suggested the contributions of cartilage and bone formation defects and increased bone resorption to bone abnormality in *Ccn2*^*fl/fl*^*;Prx1*^*Cre*^ mice. With transcriptome sequencing of BMSCs from wild-type and *Ccn2*-deficient mice, we performed pathway analysis with the differentially expressed genes (DEGs) and found that the down-regulated genes were enriched in biological pathways like multicellular organism development, collagen fibril organization, skeletal system development, and limb joint morphogenesis (Fig. S4). The RNA-seq data provide additional support suggesting that CCN2 deficiency contributed to the skeletal deformities.

## Discussion

Herein we performed genetic analysis in SEMD families without known genetic causes, and reported a monoallelic missense variant in *CCN2* (c.65 G > C, p.Arg22Pro) as the genetic cause for an autosomal dominant form of SEMD in 14 subjects. In silico analyses showed that the variant was located in the signal sequence of *CCN2* and resulted in elevated folding free energy and decreased isoelectric point of the signal peptide. And the variant showed a deleterious effect on CCN2 processing and secretion, and was potentially implicated in impaired osteogenesis. We further generated zebrafish *ccn2a* knockout model and osteoblast lineage-specific *Ccn2-*deficient mice (*Ccn2*^*fl/fl*^*;Prx1*^*Cre*^) that recapitulated the defective skeleton phenotypes observed in SEMD subjects to a certain extent, especially the phenotype of low bone mass.

CCN2 is a secretory protein consisting of an N-terminal signal peptide and four conserved modules, and is mainly secreted as an extracellular protein and interacts with a number of cell surface receptors, ECM components, and cytokines, fulling the common features as other matricellular proteins.^17–19^ Signal peptide of a secretory protein is generally short sequences in the amino terminus of newly synthesized proteins and direct the protein into the cell secretory pathway, and typically consists of three distinct and featured domains: a positively charged N-terminal region (n-region, 1–5 residues), a hydrophobic core (h-region, 7–15 residues), and a polar C-terminal region containing cleavage site (c-region, 3-7 residues).^32,33^ During the process of protein secretion, signal peptide is recognized by a targeting factor named signal recognition particle (SRP), and directs to the endoplasmic reticulum membrane for translocation, and the signal sequence is cleaved off at the luminal side of the endoplasmic reticulum membrane, and matured protein is finally transported outside the cell through Golgi.^32,33^ Several human mendelian metabolic bone disorders have been reported connected with variants in the signal sequences of pathogenic genes, such as *CTSK* for Pycnodysostosis,^34,35^ *PTH* for Hypoparathyroidism,^36^ and *COL10A1* for Schmid metaphyseal chondrodysplasia.^37^ And the pathogenic basis of the variants in signal sequences are mainly connected with defects in the secretion of mutant protein through secretory pathway or pathological degradation of the mutant mRNA through a pathway named Regulation of Aberrant Protein Production (RAPP), and these two different consequences are demonstrated to be related to the position of the variant in signal sequence.^38^ It is indicated that the n-region plays roles in the protein docking and translocation, the h-region is the target for the SRP, and c-region influences the efficiency and accuracy of signal peptide cleavage.^32,33^ Therefore, variants in the n-region can interfere with protein translocation; variants in the h-region tend to prevent the interaction with SRP, triggering pathologically activation RAPP pathway and following mRNA degradation; variants in the c-region often prevent cleavage of the signal sequence, affecting protein secretion.^38^ In this study, the identified variant (c.65 G > C, p.Arg22Pro) is located in the signal peptide of *CCN2*. In silico analyses show that the signal sequence of CCN2 consists of 26 amino acids, and the variant is located in its c-region, and elevated folding free energy and decreased isoelectric point of the signal peptide due to the variant are implicated. We find a decreased secretion and an intracellular retention of CCN2 proteins in mutant transfected cells, with the CCN2 mRNA levels similar to those of wild-type transfected cells. These indicate the potential deleterious effect of the variant on protein processing and secretion. As CCN2 mainly acts as a secretory matricellular protein in ECM, the impaired process of protein secretion is most likely responsible for the disease. Limited evidence had ever indicated that CCN2 might display some intracellular function via endocytic pathways and thus affect gene transcription in the nucleus,^39^ therefore, further studies are required to exclude the possible existence of some unidentified intracellular function resulting from accumulated mutant CCN2, which may be also involved in the pathogenic mechanisms underlying the disorder. Due to the discovery that CCN2 can be cleaved into different fragments due to the presenting of multiple cleavage sites between different modules, and different fragments of CCN2 have been indicated with different biological roles,^39,40^ thus the human phenotypes associated with variants in different modules remain to be revealed in the future.

We observe defective skeletal development among the subjects with the *CCN2* variant, including mild-to-severe disproportionate short stature with relative short lower limbs, limited joint flexion, premature osteoarthritis-like changes in weight-bearing joints, and low bone mass. CCN2 is among the group of regulatory proteins in ECM in various organs, with skeleton among the major expression sites during development.^41^ Previous in vivo studies demonstrated that CCN2 was strongly expressed in osteoblast lining metaphyseal trabeculae, an active osteogenesis site during the period of bone modeling.^42^ Through overexpressing CCN2 in BMSCs, we find that CCN2 caused an increase in ALP activity and increased mRNA levels of osteoblast-specific markers, suggesting that CCN2 lead to an enhanced osteoblast differentiation. This finding was consistent with retroviral vectors mediated CCN2 overexpression in ST-2 stromal cells, which also resulted in an increase in osteoblastogenesis.^43^ And the essential roles of CCN2 in MSCs osteogenesis was supported by findings of in vitro studies of MSCs with *CCN2* knockout, which resulted in disruption in MSCs colony formation, inhibited MSCs proliferation, and enhanced adipocytes differentiation.^44^ We show that mutant CCN2 infected BMSCs have decreased osteogenesis compared with those of wild-type, indicating that impaired osteogenesis owing to the *CCN2* variant is probably involved in the pathological mechanism underlying the SEMD. Our in vivo studies in zebrafish and mice models further provide evidence that CCN2 is required for skeletal development. *Ccn2a*-null zebrafish exhibit remarkable craniofacial skeleton dysplasia and decreased bone mass, and the abnormalities are reversed by overexpression of *ccn2a* mRNA. The zebrafish has recently emerged as a popular model for skeletal research due to the conservation of cell types in cartilage and bone, as well as the genetic pathways regulating their development between zebrafish and humans.^28,29^ Nevertheless, in zebrafish models of human disease, there is not always a one-to-one relationship in terms of the affected anatomical compartment or the degree of phenotypic severity, with potential mechanisms remaining to be elucidated.^29,45^ In present study, we did not observe notable dysmorphic facial phenotypes such as a prominent forehead, depressed nasal bridge, prominent cheekbone, cleft palate, or small jaw, as found in other certain types of SEMD, among affected subjects with CCN2 variant. However, delayed anterior fontanelle closing was observed in IV-5, and IV-4, a 15.1 year old boy, had a head circumference of 61.0 cm (normal range in adult: 54.0–58.0 cm). Currently, it remains challenging to determine whether the above findings are coincidental and to identify the potential clinical significance of our findings without more similar cases.

CCN2 has been demonstrated to display important roles in many cell types during the process of endochondral ossification and intramembranous ossification,^13,14^ two distinct processes of bone formation during embryogenesis in mammalian.^46^ Here, we also provide evidence that the requirement of CCN2 in early mesenchymal progenitor cells is potentially contributing to the molecular mechanism whereby CCN2 deficiency cause phenotypes observed in subject with SEMD. *Ccn2* deletion in mesenchymal progenitors in mice (*Ccn2*^*fl/fl*^*;Prx1*^*Cre*^), partially recapitulate the clinical findings in SEMD, cause decreased bone mass. Moreover, it was reported that the *Ccn2*^+/−^ mice also presented with osteopenia with a 30%–45% reduction in BV/TV,^47^ showing consistent phenotypes observed in *Ccn2*^*fl/fl*^*;Prx1*^*Cre*^ mice in present study. Through the immunofluorescence analysis with Col1α1, we observe decreased bone formation in metaphyseal trabeculae in *Ccn2*^*fl/fl*^*;Prx1*^*Cre*^ mice, and it is in line with the findings identified in vitro studies with MSCs. As the decreased bone mass can be the result of decreased bone formation, increased bone resorption, or a combination of both.^48^ We further examine the alternation in bone resorption using TRAP staining, and *Ccn2*^*fl/fl*^*;Prx1*^*Cre*^ mice display an increase TRAP-positive area, indicating that the bone loss is also associated with an increase in bone resorption. It was previously demonstrated that *Ccn2* knock out mice have fewer osteoclasts in the metaphyseal region.^49^ As we knock out *Ccn2* in early mesenchymal progenitor cells in present study, the increase in TRAP-positive area was suspected as a secondary alternation. The underlying mechanism of the discrepant findings and of the uncoupling of bone formation and bone resorption in *Ccn2*^*fl/fl*^*;Prx1*^*Cre*^ mice require further studies. Cartilage anabolism in growth plate determined by Col2 level through immunofluorescence analysis show that Col2-marked region is expanded in *Ccn2*^*fl/fl*^*;Prx1*^*Cre*^ mice. These results suggest that the pathological mechanism underlying the skeletal phenotypes of *Ccn2*^*fl/fl*^*;Prx1*^*Cre*^ mice involve decreased bone formation, increased bone resorption and abnormal cartilage formation. Bioinformatics analysis reveals that these down-regulated genes are enriched in some biological pathways, including multicellular organism development, collagen fibril organization, skeletal system development, and limb joint morphogenesis. The pathways enriched by KEGG pathway enrichment include cGMP-PKG signaling pathway, cAMP signaling pathway, both of which play important roles in skeletal remodeling;^50^ PI3K-Akt signaling pathway has been reported to regulate BMP2-induced MSCs osteogenic differentiation,^51^ promote fracture healing,^52^ and is essential for joint tissue metabolism and involved in the development of osteoarthritis;^53^ ECM-receptor interaction is also common in skeletal formation and regeneration through ECM dynamic interaction with osteoblasts and osteoclasts to regulate the new bone formation.^54^ And the protein-protein interaction network analysis of DEGs enrich in collagen fibril organization and skeletal system development. These data provide further evidence suggesting that CCN2 deficiency contributed to the skeletal deformities. However, it is noted that both the two mice models (*Ccn2*^*fl/fl*^*;Prx1*^*Cre*^ mice and *Ccn2*^+/−^mice) display normal femoral length, and no obvious skeletal abnormalities except for the decreased bone mass are detected. Meanwhile, we do not observe obvious abnormalities in skeletal development or homeostasis in mice harboring the identified *CCN2* variant (data not shown). CCN family have six members, and the remaining five CCN proteins have been found to be expressed during the process of bone formation and to have exhibiting similar or opposing roles to CCN2.^13,55,56^ The differences in skeletal phenotypic spectrum and severity due to CCN2 deficiency between genetic mouse models and human indicated the existence of differences in functional redundancy of CCN family members or other compensatory mechanisms for CCN2 deficiency in mice versus human. Alternatively, potential existence of differences in threshold effects of CCN2 deficiency for skeletal dysplasia between between mouse models and human remain to be clarified. The hypothesis of redundant or opposing roles of CCN proteins in skeletal development in mice is further supported by the finding that mice with *CCN6* (formerly *WISP3* [MIM: 603400]) knockout, the gene known to cause human progressive pseudorheumatoid dysplasia that is characterized by joint abnormalities, do not exhibit overt skeletal phenotypes.^57,58^ Mouse models are connected to the study of the functions of causative gene involved in human monogenic disease. However, these models may only replicate some aspects of the human disease phenotype, exhibit more severe phenotype, or have no clinical phenotype at all.^59,60^ Translating phenotypes across different species can be challenging due to potential differences in biology, genetic background, alternative pathways, or compensation mechanism.^61–65^ While the exact reasons for differences in skeletal phenotype due to CCN2 deficiency between species require further investigation, the detected discrepancies prompt us to look into the possible effects of modifier genes, alternative pathways, possible interacting proteins, and synergistic effects on the pathogenesis of SEMD, which will contribute to our understanding of biology with respect to the similarities and differences between species. In addition, despite that CCN2 has been demonstrated to display many critical functions in various physiological processes,^14,17,19^ affected subjects with CCN2 deficiency specifically exhibit skeletal abnormalities, and have no history of evident abnormalities in other organs, these indicating probable different sensitivities between skeleton and extra-skeletal organs to CCN2 deficiency in human. Although the comprehensive roles of CCN2 in human skeletal development remains to be further clarified, defective skeletal development owing to CCN2 deficiency is consistent with our understanding of pathogenesis underlying the SEMD.

In conclusion, present study therefore establish CCN2 as a pathogenic gene to SEMD spectrum, and uncover the human phenotypes causally associated with CCN2 deficiency. Given the process of skeletal development is potentially sensitive to CCN2 dosage reduction, the evaluation of skeleton alternations in subjects with CCN2-target therapies is potentially necessary in clinical settings. Further studies are required and expected to determine whether modulating CCN2 could provide a new therapeutic approach for osteoporosis.

## Materials and methods

### Study approval

The human study was conducted in accordance with the principles of the Declaration of Helsinki, and was approved by the Ethics Committee of the Shanghai Jiao Tong University of Medicine Affiliated Sixth People’s Hospital. Written informed consent was received from participants or parents of minors prior to inclusion in the study. The zebrafish experiments were approved by the Institutional Animal Care and Use Committee of Shanghai Research Center for Model Organisms. And the zebrafish facility is accredited by the Association for Assessment and Accreditation of Laboratory Animal Care (AAALAC) International. The mice were bred and maintained under specific pathogen free conditions in the institutional animal facility of the Shanghai Institute of Biochemistry and Cell Biology, Chinese Academy of Sciences. All mice experiments were conducted in accordance with a protocol approved by the Animal Care and Use Committee of Shanghai Institute of Biochemistry and Cell Biology, Chinese Academy of Sciences.

### Clinical and genetic analysis

#### Human subjects and clinical analysis

We investigated probands with SEMD and their available family members. All individuals underwent a thorough clinical inquiry, physical examination, biochemical and bone-densitometry analyses, and skeletal radiography. The diagnosis of SEMD was based on clinical manifestations and radiological findings. In addition, 750 healthy unrelated Chinese subjects were enrolled as controls.

#### Genome-wide linkage analysis

Genomic DNA was extracted and purified from peripheral blood leukocytes with traditional methods. Genotyping of the samples was performed using Illumina’s GenomeStudio Genotyping Module, with data collected according to Infinium HD Assay Super protocol. Multipoint parametric linkage analysis and haplotyping for a dominant inheritance model with complete penetrance were conducted by the MERLIN program, version 1.1.2. Putative protein-coding genes within candidate region were examined using UCSC Genome Browser on human Feb. 2009 (GRCh37/hg19) assembly (http://genome.ucsc.edu/).

#### Whole-exome sequencing

Qualified DNA sample was fragmented, ligated to adapters, amplified by ligation-mediated PCR, and hybridized to exome array for enrichment. Agilent 2100 Bioanalyzer and quantitative PCR were used to estimate the magnitude of enrichment of captured ligation-mediated PCR products. High-throughput sequencing were conducted for each qualified capture library loaded on Illumina Hiseq platforms. The derived data were then processed by Illumina base-calling Software for base-calling with default parameters, and were generated as paired-end reads. High quality reads were mapped to the human reference genome GRCh37 with Burrows-Wheeler Aligner (BWA V0.7.15), and duplicate reads were removed by Picard-tools (v2.5.0). Genome Analysis Toolkit (GATK) was used for local realignment around InDels and base quality score recalibration following best practices. HaplotypeCaller of GATK (v3.3.0) was applied for detection of genomic variation including SNPs and InDels, and high-confident variant calls were obtained using hard-filtering methods. SnpEff tool was conducted for a series of annotations for variants. Variants with an allele frequency >0.1% in the 1 000 Genomes Project database (http://www.1000genomes.org/), the ESP6500 (http://evs.gs.washington.edu/EVS/), and the ExAC database (http://exac.broadinstitute.org/) were excluded.

#### Sanger sequencing

The variants in candidate pathogenic genes, identified by combination of linkage analysis and WES, was confirmed by Sanger sequencing following PCR amplification in all available family members. Amplificated PCR products were purified with shrimp alkaline phosphatase (Promega) and exonuclease I (Epicentre). Sequencing reaction was performed using BigDye Terminator cycle sequencing ready reaction kit, version 3.1 (Applied Biosystems), and the products were detected and analyzed using an ABI Prism 3130 automated sequencer (Applied Biosystems) and Polyphred.

### Cell culture

Mice BMSCs were isolated from tibia and femur bone marrow cavity of 4-week-old C57BL/6 mice: bone marrow was flushed out with pre-cooled PBS, followed by centrifugation and lysis of erythrocytes. The cell precipitates were gently resuspended and cultured in α-MEM supplemented with 10% FBS and 1% penicillin/streptomycin. HEK293T cells and C3H10T1/2 cells were purchased from the American Cell Type Culture Collection (ATCC), and HEK293T cells were cultured in DMEM containing 10% FBS and 1% penicillin/streptomycin, C3H10T1/2 cells were cultured in α-MEM containing 10% FBS and 1% penicillin/streptomycin. All the cells were cultured in a 5% CO_2_ humidified incubator at 37 °C.

### Transfection and infection

Phage vector overexpressing Flag-tagged full-length human mutant CCN2 (p.Arg22Pro) or wild-type CCN2 were constructed. HEK293T cells were transfected with the wild-type or mutant vector together with pLenti-EGFP according to the Effectene Transfection protocol (QIAGEN, 301425), with pLenti-EGFP as a control. For lentivirus vector construction, we overexpressed ZsGreen, Flag-tagged full-length human mutant CCN2 or wild-type CCN2 using a pLenti vector (pLenti-hCCN2-3xFlag-ZsGreen-Puro). Lentivirus packaging was conducted according to the VSVG-delta 8.9 system. BMSCs or C3H10T1/2 were infected with lentivirus for 24 h, and treated with puromycin for 48 h. The ZsGreen lentivirus was used as a control.

### In vitro osteoblast differentiation

For in vitro osteoblast differentiation, BMSCs treated with lentivirus were induced by α-MEM with 10% FBS, 1% penicillin/streptomycin, 50 μg/mL L-ascorbic acid (Sigma, A5960), and 1.08 mg/mL β-Glycerophosphate disodium salt hydrate (Sigma, G9422). On day 7, BMSCs were fixed with 4% paraformaldehyde (PFA) for 10 min and subjected to ALP staining according to the manual of BCIP/NBT Alkaline Phosphatase Color Development Kit (Beyotime, C3206). For ALP activity quantitative analysis, osteoblasts were incubated with Alamar Blue (Invitrogen) for 1 h and read with a luminometer (Envision) for cell number quantification. Then the osteoblasts were incubated with phosphatase substrate (Sigma, S0942) dissolved in the solution (6.5 mmol/L Na_2_CO_3_, 18.5 mmol/L NaHCO_3_, 2 mmol/L MgCl_2_) for 20 min. The ALP activity was read with a luminometer (Envision) at A405, and normalized as A405/Alamar Blue.

### Protein secretion analysis

For CCN2 secretion analysis, HEK293T cells were transfected with indicated plasmids. Cells were harvested after 48 h and cells were lysed with 500 μL EBC buffer (50 mmol/L Tris, pH 7.5, 120 mmol/L NaCl, and 0.5% NP-40) supplemented with a protease inhibitor cocktail (MCE, HY-K0010) and centrifuged at 12 000 r/min. Culture medium was collected into 15 mL centrifuge tube and centrifuged at 2 000 r/min. Cell lysates and medium were analyzed by sodium dodecyl sulfate polyacrylamide gel electrophoresis (SDS-PAGE) and immunoblotted with primary antibody against Flag (Sigma, F3165-5MG, 1:5 000) or GFP (Proteintech, 66002-1-Ig, 1:5 000) overnight at 4°C and secondary antibody of anti-mouse-immunoglobulins/HRP (Dako, P0260). After incubation with specific antibodies, we used enhanced chemiluminescence kit (Millipore) to detect the protein signals. Our method of quantification is to do a ratio between the destination bands and their matching internal reference bands after quantification. The ratio of each destination band is then normalized and presented below each destination band.

### Immunofluorescence

For cell immunofluorescence analysis, C3H10T1/2 cells infected with indicated plasmids after 48 h were fixed in 4% PFA for 15 min and blocked in PBS with 3% horse serum and 0.3% Triton X-100 (Sangon Biotech, A110694-0500) for 30 min, then incubated with mouse-anti-Flag (Sigma, F3165-5MG, 1:200) overnight at 4 °C. Donkey anti-mouse-Cy3 (Jackson ImmunoResearch, 715-165-150, 1:1 000) was used as secondary antibody. DAPI (Sigma, D8417, 1:1 000) was used for counterstaining. Cells were mounted with anti-fluorescence mounting medium (Dako, S3023) and images were captured with a Leica SP8 confocal microscope.

### RT-PCR analysis

Total RNA of cells was isolated in Trizol (Invitrogen) and reverse transcribed into cDNA using Transcriptor First strand cDNA synthesis kit (Roche). RT-PCR analysis was conducted with FastStart Universal SYBR Green Master (Roche) in a LightCycler480 PCR system (Roche). Relative expression of target genes was normalized to Gapdh or Hprt, and was calculated with delta-delta CT method. The primer used are listed as follows: *hCCN2*-qF, 5’-AAGGGCAAAAAGTGCATCCG-3’; *hCCN2*-qR, 5’-TCTTCTTCATGACCTCGCCG-3’; *mGAPDH*-qF: 5’-TTCCTACCCCCAATGTGTCC-3’; *mGAPDH*-qR, 5’-GGTCCTCAGTGTAGCCCAAG-3’; *mAlp*-qF, 5’-CGGGACTG GTACTCGGATAA-3’;*mAlp*-qR, 5’-ATTCCACGTCGGTTCTGTTC-3’; *mCcn2*-qF, 5’-CTGCAGGCTAGAGAAGCAGAG-3’; *mCcn2*-qR, 5’-GATGCACTTTTTGCCCTTCT-3’; *mRunx2*-qF, 5’-CCAACCGAGTCATTTAAGGCT-3’; *mRunx2*-qR, 5’-GCTCACGTCGCTCATCTTG-3’; *mBglap*-qF, 5’-GCAGCACAGGTCCTAAATAG-3’; *mBglap*-qR, 5’-GGGCAATAAGGTAGTGAACAG-3’; *mHprt*-qF, 5’-GTTAAGCAGTACAGCCCCAAA-3’; *mHprt*-qR, 5’-AGGGCATATCCAACAACAAACTT-3’.

### Human serum CCN2 levels measurement

CCN2 protein levels in human serum were determined using Elisa kits (DRG Instruments GmbH, EIA-5195) following the manufacturer’s instructions. The intra-assay coefficients of variation (CVs) for CCN2 were 1.3% at a level of 110.1 ng/mL, 2.6% at a level of 49.9 ng/mL, 3.9% at a level of 129.7 ng/mL. The inter-assay CVs for CCN2 were 9.7% at a level of 27.2 ng/mL, 9.8% at a level of 15.0 ng/mL, 10.6% at a level of 31.0 ng/mL, 6.2% at a level of 32.2 ng/mL.

### *CCN2* knock-down studies in Zebrafish

#### Zebrafish care, maintenance, and microinjections

Adult wild-type zebrafish (AB strain) were maintained at 28.5 °C on a 14 h light/10 h dark cycle,^66^ and 5 to 6 pairs of them were set up for nature mating every time. The generated embryos were maintained at 28.5 °C in fish water (0.2% Instant Ocean Salt in deionized water), and were washed and staged according to previous studies.^67^ For *ccn2a* gene knock-down experiment, the *ccn2a*-e2i2-MO (5’-AACAGCCAAGATCCTTACCTGTGCA-3’) and the standard control MO (5’-CCTCTTACCTCAGTTACAATTTATA-3’), was designed (Gene Tools, http://www.gene-tools.com/) and microinjected into fertilized one-cell stage embryos.^68^ RT-PCR analysis and Sanger sequencing were performed to confirm the efficacy of the *ccn2a*-e2i2-MO. Total RNA of 30 to 50 embryos per group was extracted and reverse transcribed into cDNA. The primers of *ccn2a* spanning exon 1 and exon 3 were 5’-CTCTGCTGTTCCTGACTTTCTT-3’ (forward) and 5’-ACTTCCCAGGCACTTTCAC-3’ (reverse). The primers of *ef1α*, used as the internal control, were 5’-GGAAATTCGAGACCAGCAAATAC-3’ (forward) and 5’-GATACCAGCCTCAAACTCACC-3’ (reverse). For rescue experiments, 4 ng *ccn2a*-e2i2-MO was co-injected with 100 pg pcDNA3.1 containing nonmutant zebrafish *ccn2a* cDNA per embryo. The coding region of the wild-type zebrafish *ccn2a* was synthed by Sangon Biotech and subcloned into pcDNA3.1 vector (Invitrogen).

#### Calcein staining and image acquisition

Zebrafish at 5-dpf were firstly washed with fish water and immersed in 0.2% calcein solution for 10 min. And the zebrafish were then rinsed thoroughly in fish water again and anaesthetized with 0.016% MS-222 (tricaine methanesulfonate, Sigma-Aldrich). Finally, the zebrafish were oriented on ventral side and mounted with 3% methylcellulose (Sigma-Aldrich) in a depression slide for observation by fluorescence microscopy (Nikon SMZ 1500) and subsequently photographed with digital cameras. Qualification of the RFI of head skeleton bone mass was performed using morphometric analysis (NIS-Elements D3.1). To optimally visualize the expression patterns, a subset of images was adjusted for levels, brightness, contrast, hue and saturation (Adobe Photoshop 7.0 software). A total of 10 zebrafish for each group were quantified and averaged.

#### Ccn2^fl/fl^;Prx1^Cre^ mice model analysis

##### Mice model generation

*Ccn2*^*fl/fl*^ mice bearing loxP sites flanking exons 1–5 of the *Ccn2* gene were provided by GemPharmatech (Nanjing, China). The *Prx1*^*Cre*^ mice strain was a gift from Andrew McMahon. *Ccn2*^*fl/fl*^ mice were crossed with *Prx1*^*Cre*^ mice to generate *Ccn2*^*fl/fl*^*;Prx1*^*Cre*^ mice. All mice analyzed were maintained on the C57BL/6 background.

##### Micro-CT analysis

Left femurs isolated from age- and sex-matched 6-week-old *Ccn2*^*fl/fl*^*;Prx1*^*Cre*^ mice and control littermates were fixed in 70% ethanol and scanned using SkyScan-1176 micro-computed tomography (μCT) (Bruker micro CT, Belgium) system. Scans were performed using 8.96 μm voxel size, 50 KV, 500 μA and 0.4 degrees rotation step (180 degrees angular range). The 1.6 version of NRecon software (Bruker) was used for three-dimensional (3D) reconstruction and viewing of images, and 3D photos of bones were performed using 3.3.0 version of CTvox software (Bruker). After 3D reconstruction, the 1.13 version of CTan software (Bruker) was used for bone analysis, and the segmentation were range from 85/120 to 255/255. For trabecular bone, micro-CT evaluation was performed on a 2 mm region of metaphyseal spongiosa in the distal femur, located 0.5 mm above the growth plate. For cortical bone, measurements were performed on a 0.5 mm region of the mid diaphysis of the femur. Trabecular bone measurements included BMD, BV/TV, Tb.Th, Tb.N and Tb.Sp. Cortical bone measurements included Ct.Th.

##### Histological staining, immunofluorescence, and immunohistochemistry

Femur and tibiae bones from mice were fixed with 4% PFA for 48 h at 4 °C, decalcified in 10% EDTA for 2 weeks, dehydrated in alcohol, cleared with xylene, and embedded in paraffin. Each sample was sectioned sagittally using microtome (Lexica Microsystems Nussle GmbH) at a thickness of 8 μm for staining. H/E staining were performed using a standard protocol. For TRAP staining, sections were stained with the Leukocyte Acid Phosphatase Kit (Sigma, 387A-1KT) for 1 h at 37 °C following the manufacturers’ instructions. For immunofluorescence, sections were blocked in PBS with 10% horse serum and 0.3% Triton X-100 (Sangon Biotech, A110694-0500) for 1 h and then stained overnight at 4 °C with rabbit-anti-Collagen II (Abcam, ab34712) or rabbit-anti-Collagen I (Abcam, ab21286). Secondary antibodies were used according to the species of the primary antibody, and DAPI (Sigma, D8417) was used for counter staining. Slides were mounted with anti-fade fluorescence mounting medium (Dako, S3023). Images were acquired with microscope (Olympus BX51, Tokyo, Japan).

### RNA sequencing and analysis

We collected femurs and tibias of wild-type and *Ccn2*-deficient mice, removed the surrounding soft tissues, and isolated BMSCs: bone marrow was flushed out with pre-cooled PBS, followed by centrifugation and lysis of erythrocytes. The cell precipitates were gently resuspended in culture medium (α-MEM supplemented with 10% FBS and 1% penicillin/streptomycin), seeded (2 × 10^6^ cells per dish) in 100-mm culture dishes, and incubated at 37 °C under 5% CO_2_ conditions. After 48 h, nonadherent cells were washed away with PBS, adherent cells were cultured for another 5 days. We collected the cells and obtained RNA for transcriptome sequencing (fold change greater than 2.0 and *P* value less than 0.05). The data were analyzed on the free online platform of Majorbio Cloud Platform (www.majorbio.com).

### Statistics

Statistical analysis was performed using the GraphPad Prism 9 software (GraphPad Software). Cell-based experiments were performed at least twice. Animals were randomized into different groups and at least 3 mice were used for each group, unless otherwise stated. All quantitative data are presented as mean ± SD. Student’s *t* test, one-way ANOVA, and Mann–Whitney test was used, as appropriate, for statistical evaluations of group comparisons. Statistical significance is indicated by *, where *P* < 0.05, **, where *P* < 0.01, ***, where *P* < 0.001, ****, where *P* < 0.000 1, and all tests were two-sided.

## Supplementary information

BONERES-03199R3. Revised Supplementary Appendix_Shanshan Li

## Data Availability

Data that support the findings of this study are present in the paper and/or the Supplementary Materials, and additional data related to this paper are available from the corresponding author upon reasonable request.
